# Increased endocardial to epicardial flow ratio present at rest disappears during exercise stress perfusion CMR in normal volunteers - a potential mechanism for exercise induced subendocardial ischaemia

**DOI:** 10.1186/1532-429X-14-S1-P226

**Published:** 2012-02-01

**Authors:** Kaleab N Asrress, Rupert Williams, Tim Lockie, John D Biglands, Amedeo Chiribiri, Aleksandra Radjenovic, Roy Jogiya, Kalpa De Silva, Sebastian Kozerke, Phil Chowienczyk, Eike Nagel, Michael Marber, Simon Redwood, Sven Plein

**Affiliations:** 1 Cardiovascular Sciences, King's College London, London, UK; 2 Imaging Sciences, King's College London, London, UK; 3 Multidisciplinary Cardiovascular Research Centre, University of Leeds, Leeds, UK; 4 Division of Medical Physics, University of Leeds, Leeds, UK

## Background

Exercise stress testing remains the most physiological method of inducing myocardial stress. The feasibility of physiological stress in the MR environment have limited its clinical and scientific application, but MR compatible ergometers are now available that permit measurement of the response to exercise-stress. An important mechanism governing inducible ischaemia is the redistribution of myocardial blood flow between the endocardial and epicardial layers. With high-resolution CMR methods, these responses can be measured providing a potential technique with which these parameters can be studied during physiological stress.

The purpose of this study was to quantify absolute myocardial blood flow as well as endocardial and epicardial segmental flow during rest and exercise stress; and to demonstrate the reproducibility of these measurements under serial exercise.

## Methods

Healthy volunteers without known cardiovascular disease gave informed consent for supine cycle ergometry on the CMR scanner table (Lode, Netherlands). Subjects underwent a standardised incremental exercise protocol. High-resolution perfusion CMR was performed on a 3T Philips Achieva® system using 0.04 mmol/kg Gd-DTPA (saturation recovery gradient echo, repetition time/echo time 2.7ms/0.96ms, flip angle 15°, 5 x k-t SENSE acceleration, 11 interleaved training profiles, spatial resolution 1.0x1.0x8mm3, 3 slices acquired at each RR interval, 30 dynamic images). After a period of rest for 20 min, the exercise protocol was repeated with a further perfusion scan.

Data were analysed with MASS® (Medis, Netherlands) and Matlab® (The MathWorksInc®, USA). Subendocardial and subepicardial borders were traced and the LV myocardium was divided into 6 segments per slice and their endocardial, epicardial and middle thirds. Transmural myocardial blood flow (ml/g/min), as well as endocardial and epicardial flow ratios were estimated using Fermi-function derived deconvolution. Data are presented as mean ±SEM.

## Results

Seven subjects (mean age 36.0 years) completed two periods of exercise. Resting transmural blood flow was 0.8±0.06 ml/g/min that increased to 2.6±0.4 and 2.5±0.3 ml/g/min during exercise 1 and 2 respectively (p=0.001 rest vs exercise). Myocardial perfusion reserve was 3.3±0.5 and 3.1±0.4 respectively (non-significant). There was an endocardial:epicardial flow ratio at rest of 1.2±0.06 which reduced to 1.05±0.08 after exercise 1 and 1.07±0.08 after exercise 2 (p = 0.01 vs rest).

## Conclusions

High-resolution myocardial perfusion CMR demonstrated changes in endocardial and epicardial myocardial blood flow during cycle ergometer stress. Serial exercise provides reproducible perfusion quantification. The loss of the resting endocardial:epicardial blood flow ratio during exercise stress is a potential mechanism for exercise induced endocardial ischaemia in patients with coronary artery disease.

## Funding

The authors are supported by Clinical Research Training Fellowships from the British Heart Foundation; as well as the Department of Health via the National Institute for Health Research comprehensive Biomedical Research Centre award to Guy’s and St Thomas’ NHS Foundation Trust.

